# High glucose causes developmental abnormalities in neuroepithelial cysts with actin and HK1 distribution changes

**DOI:** 10.3389/fcell.2022.1021284

**Published:** 2023-01-06

**Authors:** Sisi Peng, Yu Wu, Yufang Zheng

**Affiliations:** ^1^ Department of Cellular and Developmental Biology, School of Life Sciences, Fudan University, Shanghai, China; ^2^ Obstetrics and Gynecology Hospital, The Institute of Obstetrics and Gynecology, Fudan University, Shanghai, China; ^3^ State Key Laboratory of Genetic Engineering, School of Life Sciences, Fudan University, Shanghai, China

**Keywords:** high glucose, actin, HK1, neuroepithelial cysts, neural rosettes

## Abstract

It has been reported that the offspring of diabetic pregnant women have an increased risk for neural tube defects. Previous studies in animal models suggested that high glucose induces cell apoptosis and epigenetic changes in the developing neural tube. However, effects on other cellular aspects such as the cell shape changes were not fully investigated. Actin dynamics plays essential roles in cell shape change. Disruption on actin dynamics is known to cause neural tube defects. In the present study, we used a 3D neuroepithelial cyst model and a rosette model, both cultured from human embryonic stem cells, to study the cellular effects caused by high glucose. By using these models, we observed couple of new changes besides increased apoptosis. First, we observed that high glucose disturbed the distribution of pH3 positive cells in the neuroepithelial cysts. Secondly, we found that high glucose exposure caused a relatively smaller actin inner boundary enclosed area, which was unlikely due to osmolarity changes. We further investigated key glucose metabolic enzymes in our models and the results showed that the distribution of hexokinase1 (HK1) was affected by high glucose. We observed that hexokinase1 has an apical-basal polarized distribution and is highest next to actin at the boundaries. hexokinase1 was more diffused and distributed less polarized under high glucose condition. Together, our observations broadened the cellular effects that may be caused by high glucose in the developing neural tube, especially in the secondary neurulation process.

## Introduction

Neural tube defects (NTDs) are among the most common birth defects in humans, which originated from abnormal neural tube development during early embryonic development. Both genetic and environmental factors can cause NTDs (Copp et al., 2013; Copp and Greene, 2013; Wallingford et al., 2013; Greene and Copp, 2014). One of the environmental factors is maternal diabetes. Clinical studies reported the offspring of diabetic pregnant women have a higher NTD risk (Becerra et al., 1990; Correa et al., 2008). Studies in animal models suggested that maternal hyperglycemia is teratogenic and can produce NTDs in exposed offspring (Zabihi and Loeken, 2010). Studies in animal models also suggested that at the cellular level, increased cellular stress and apoptosis is involved in the etiology of NTDs secondary to high maternal glucose (Wu et al., 2015; Loeken, 2020). Cellular studies showed that high glucose can change many aspects in neural stem/progenitor cells, including suppressing neural differentiation (Yang et al., 2016; Chen et al., 2018), altering DNA methylation pattern (Shyamasundar et al., 2013; Kandilya et al., 2020), histone acetylation (Shyamasundar et al., 2013; Yang et al., 2016; Bai et al., 2018), and even miRNA expression (Shyamasundar et al., 2013; Ramya et al., 2017).

At the present time, there is data gap with respect to possible cell shape changes caused by high glucose in the neural tube. The neural tube undergoes extensive shape changes during development, and actin plays an important role in shape changes during neuralation (Suzuki et al., 2012; Nikolopoulou et al., 2017). Actin is largely regulated by actin-binding proteins (Pollard, 2016). Myosin II and cofilin (CFN) are two actin-binding proteins known to regulate actin organization during neural tube development (Nikolopoulou et al., 2017). Myosin II is an actin motor protein that helps cargo movements of actin and can lead to actin contraction (Sweeney and Holzbaur, 2018). The main function of CFN is to sever actin (Pollard, 2016). Both proteins are regulated by their phosphorylation state. While phosphorylation of myosin light chain (MLC) activates myosin II (Matsumura, 2005), phosphorylation inactivates CFN (Mizuno, 2013). Disrupting actin related proteins by genetic modification can cause NTDs in animal models (Copp et al., 2013). For example, genetic depletion of CFN1, the non-muscle CFN, can lead to both cranial and spinal NTDs in the mouse (Escuin et al., 2015). Non-genetic disturbance of actin by chemical reagents can also cause NTDs in animal models. Blebbistatin, a myosin II inhibitor, when added externally, can induce NTD phenotypes in both chick and mouse embryos. 100 μM Blebbistatin can cause both anterior and posterior NTDs in the chick (Kinoshita et al., 2008), while 50 μM blebbistatin caused only cranial NTDs in the culture mouse embryos (Escuin et al., 2015). It has been known that MLC is phosphorylated by Rho kinase (ROCK) (Kimura et al., 1996); and ROCK can also activate LIM domain kinase (LIMK), which consequently phosphorylate CFN (Maekawa et al., 1999). Y27632 is a selective inhibitor of ROCK and exposure to 50 μM Y27632 caused both anterior and posterior NTDs in the chick (Kinoshita et al., 2008). Treatment with 5 μM Y27632 caused both cranial and spinal NTDs in the mouse (Escuin et al., 2015). Actin polymerization can also be interrupted by chemical reagents such as cytochalasin D (CD). In the chick, 1 μM CD can cause severe NTDs at all anterior-posterior levels, and .2 μM CD prevented normal neural tube closure at the forebrain and spinal cord levels (Kinoshita et al., 2008). Meanwhile, .05 μg/ml (∼0.1 µM) CD induced cranial but not spinal NTDs in the mouse (Escuin et al., 2015).

High glucose can also affect actin dynamics as several previous studies reported, and the effects varied on different cell types. For examples, high glucose can increase F-actin intensity in hepatocellular carcinoma cells (Topel et al., 2021) but caused only actin disorganization in podocytes (Xu et al., 2020). Whether high glucose can disrupt actin in neural tube development remains unknown and should be further investigated. In addition, high glucose can broadly affect multiple metabolic processes during neural tube development as was recently reviewed (Loeken, 2020). High glucose increases glucose import, oxidative phosphorylation, and the hexosamine biosynthetic pathway (HBSP) flux, but inhibits pentose phosphate pathway (PPP) (Loeken, 2020). It has long been known that actin cytoskeleton directly binds metabolic enzymes, including the glycolytic enzymes PFK1 and GAPDH (Knull and Walsh, 1992; DeWane et al., 2021). Considering that actin remodeling is a high-energy consuming process, it is possible that high glucose may affect actin through carbohydrate metabolic enzymes.

The formation of neural tube starts from neurulation process, which consists of two phases, primary and secondary neurulation. In primary neurulation, the neural plate folds and invaginates into the body and separates from the surface ectoderm to form an underlying hollow tube; while aggregates of mesenchyme cells form a solid cord which undergoes a mesenchymal-epithelial transition and forms cavities in secondary neurulation. Most vertebrates undergo primary neurulation in the anterior region and secondary neurulation in the posterior region around the tail bud. Failure in either neurulation processes could lead to NTDs with different phenotypes, for example anencephaly and spina bifida occulta respectively (Copp et al., 2013; Copp and Greene, 2013; Nikolopoulou et al., 2017; Ravi et al., 2021). Although maternal diabetes has been associated with spina bifida as well (Mowla et al., 2020; Forci et al., 2021), little is known about the effect of high glucose on secondary neurulation.

To study the effect of high glucose concentrations on actin during secondary neurulation, we used a three dimensional (3D) neuroepithelial cyst model (Zhu et al., 2013) and a rosette model (Shi et al., 2012a; Shi et al., 2012b) It has been suggested that the rosette model is reminiscent of secondary neurulation instead of primary neurulation (Fedorova et al., 2019). The recently developed 3D neuroepithelial cyst model is organoid-like model, which is a good *in vitro* model to study the cellular events of human neural tube development (Wu et al., 2021). Therefore, we used both models in our study. Besides increased apoptosis, our results showed that the apical location of pH3 (phosphorylated H3, a mitotic marker) positive cells was disrupted under the high glucose condition. Furthermore, we observed that actin was concentrated at the apical and basal edges in these models, and thus formed an inner and an outer boundary respectively. High glucose could cause a relatively smaller actin inner boundary enclosed area which was unlikely due to osmolarity effect. We found that Hexokinase1 (HK1) also has apical-basal distribution and it was next to the boundary actin but not co-localized. High glucose could cause a change in the HK1 distribution in the 3D model, which may associate with a reduced mitochondrial location of HK1.

## Materials and methods

### Human embryonic stem cell culture

The human embryonic stem cell line H9 (gift from Dr. Hongyan Wang’s Lab, Fudan University, Shanghai, China) was used in our experiment. Cell attachment was achieved using Matrigel^®^ (Corning, 354277) with a dilute factor half of that suggested in the certificate of each lot. Culturing medium was mTeSR™1 medium (STEMCELL). Cells were passaged by using PSCeasy^®^ human pluripotent stem cell digest (Cellapy^®^). Cell freezing medium was a mix of 10% (v/v) DMSO, 30% (v/v) Knockout™ Serum Replacement (Gibco), 60% (v/v) mTeSR™1 medium.

### 3D neuroepithelial cyst model culture

3D neuroepithelial cyst model is cultured based on a published method (Zhu et al., 2013). When H9 cells reached a growth confluence of 50%–60%, the cells from one well of the 6-well plate were digested and collected in a centrifuge tube. After centrifugation, the supernatant was aspirated as much as possible. Then 400 μl Matrigel^®^ was added to cells and mixed gently. Every 32 μl of Matrigel^®^ mixed with cells was added on a 9 mm cell slide in one well of the 48-well plate. The plate was then placed in the 37°C, 5% CO_2_ incubator for 10 min. After this, filter-sterilized N2B27 medium was added, which consisted of 20 ml Neurobasal™ Medium (Gibco), 20 ml DMEM/F-12 (Gibco), 200 μl GlutaMAX™ Supplement (Gibco), 200 μl MEM Non-Essential Amino Acids Solution (100X) (Gibco), 200 μl N-2 Supplement (100X) (Gibco), and 200 μl B-27™ Supplement (50X), serum free (Gibco). The medium was changed every day. The fifth day was the endpoint of the neuroepithelial cyst culture.

### Rosette model culture

Rosette model is also based on a published method (Shi et al., 2012a; Shi et al., 2012b). When the H9 cells in the 6-well plate grow to ∼95% confluence, the mTeSR™1 medium was replaced with the filter-sterilized 3NM medium, which consisted of 40 ml N2B27 medium, .4 μmol SB-431542 (MCE), .04 μmol Dorsomorphin (MCE), and 100 μg Insulin (human) (MCE). The medium was changed every day. After 8–10 days (when an obvious thickened and whitened culture layer can be observed with the naked eyes), the cells were passaged. Cells from one well of the 6-well plate were equally divided into 24 wells of a 48-well plate. 9 mm cell slides (required for confocal microscopy imaging) were placed in the 48-well plate before cells are seeded, and wells were coated with Matrigel^®^, which is diluted as in the embryonic stem cell culture. One day after passage, the medium was changed to N2B27 medium supplemented with 20 ng/ml Recombinant Human FGF-basic (154 a.a.) (PEPROTECH), and the medium was changed every day. The fourth day after passage was the endpoint of the rosette culture.

### High glucose setting

The normal glucose concentration of the medium was 13 mM for mTeSR™1 medium, 21.25 mM for 3NM medium and N2B27 medium. 2x glucose concentration was used as the high glucose condition in our experiments. The high glucose condition was achieved by adding D-glucose (stock solution 1 M) to the normal medium until the glucose concentration was twice the normal concentration, that is mTeSR™1 medium with 26 mM glucose and N2B27 medium with 42.5 mM glucose. Equimolar mannitol was used for osmolarity control, which means that 13 mM mannitol was added to mTeSR™1 medium and 21.25 mM mannitol was added to N2B27 medium to compare with the 2X glucose condition.

### Immunofluorescence

The samples were fixed in 4% PFA, 20 min for the neuroepithelial cyst models and 10 min for the rosette models. The blocking buffer was prepared by 5% (v/v) goat serum and .3% (v/v) triton X-100 in PBS. The primary antibodies were diluted in the blocking buffer according to the dilution ratios (Supplementary Table S1). The diluted primary antibody was added to the fixed and washed samples and incubated at 4°C overnight. The secondary antibodies were diluted in the blocking solution according to the dilution ratios (Supplementary Table S1). The diluted secondary antibody was added to each sample and incubated at 4°C overnight. The samples were washed with PBS after each antibody incubation. The rosettes were washed 3 × 4min and the 3D cysts were washed 3 × 7 min. Mounting media was the ProLong™ Glass Antifade Mountant (Invitrogen). Confocal imaging was performed using a Zeiss LSM700 microscope.

### qRT-PCR

Total RNA was extracted from the neuroepithelial cysts with TRIzol reagent (Invitrogen) and chloroform, precipitated with isopropanol, washed by 70% RNase-free ethanol and reverse transcribed into cDNA. 1 μg RNA were used to reverse to cDNA with the ReverTra Ace^®^ qPCR RT Mater Mix (Code No.FSQ-301) according to the manufacture’s protocol. Then 10 ng cDNAs products were used for qRT-PCR experiments with SYBR^®^ Green Realtime PCR Master Mix (Code No.QPK-201) and performed on the LightCycler^®^ 480 Ⅱ Real-Time PCR System (Roche). Relative expression of each target was calculated with human *ACTB* (which encodes beta-Actin) as a benchmark based on the delta Ct method. All qRT-PCR experiments were carried out in at least three replicates. Comparison between samples was performed using a Student’s *t* test. A *p*-value < .05 was considered statistically significant. The primers used in the qRT-PCR assays are listed in Supplementary Table S2.

### Graphical quantification and statistical analysis

The number of pH3-positive dividing nuclei was counted manually. In the 3D model image, the number of pH3-positive cells was counted per single cyst. In the rosette model image, the number of pH3-positive cells was counted within each actin outer boundary.

The size of interested areas were measured in the ImageJ software using functions including “color threshold,” “selection brush tool,” and “measure.” The interested areas included (A) actin inner boundary enclosed area of a single model, (B) actin outer boundary enclosed area of a single model, (C) the total CASP3 positive area in the 3D cyst, (D) the total CASP3 positive area excluding the part within actin inner boundary of a single 3D cyst, (E) the total CASP3 positive area excluding the part within actin outer boundary of a single 3D cyst, and (F) total CASP3 positive area in a fixed rosette. And the CASP3 density was calculated as: the inner part density of a 3D cyst =(C-D)/A, the outer part density of a 3D cyst =(D-E)/(B-A), and the density in a rosette = F.

The *t*-test was used in the hypothesis test when the data from the two glucose concentration groups were both normally distributed, otherwise, the Wilcoxon rank-sum test was used. The repeats of objects per condition (N/condition) used for statistical analysis are shown in legends. For most experiments, batch number was 2–4. Two to three wells of repeat per condition were included in each batch. Multiple objects per well were measured in each well. Therefore, the number of objects used in statistical analysis equal to the number of objects measured in each batch multiplied by the number of batches repeated. Statistical graph shows data points and mean ± SD. A *p*-value < .05 was considered statistically significant.

The quantification of HK1 and TOM20 distribution was also performed using the ImageJ software. An area ranging from the apical side to the basal side was chosen by the “rectangle” function. The “plot file” function was then utilized. Each object was measured twice in two different selected areas. The data series was saved, transformed to a same length, and scaled to a range of 0–1 by dividing the peak value. The repeats of objects per condition (N/condition) used for statistical analysis are shown in legends. Points and filled areas in the figure show mean ± SD.

## Results

### Effects of high glucose on model neural tube development characteristics

The 3D neuroepithelial cyst model (Zhu et al., 2013) and the rosette model (Shi et al., 2012a; Shi et al., 2012b) used in our study are based on previous reports. In the 3D neuroepithelial cyst model, the neural induction stage took 5 days after the stem cell culture was initiated. As early as day 1 (D1), cell clusters embedded in the Matrigel rearranged to form cystic shape organoids. In the rosette model, the neural induction stage took 8–10 days. After that, the cells were passaged to a new dish in which they will form rosette structures within the next 4 days. As shown before, both models can represent some aspects of the neural tube development, including apical-basal polarity and neural progenitor differentiation. Therefore, we used both models in this study. The normal glucose concentration of the medium was 13 mM for mTeSR™1 medium, 21.25 mM for 3NM medium and N2B27 medium, which are already higher than the physical condition. To observe the effect of high glucose, we used 2x glucose concentration as the high glucose condition in our experiments. The high glucose condition was achieved by adding D-glucose (stock solution 1 M) to the normal medium until the glucose concentration was twice the normal concentration.

The *in vivo* neural tube development has three major characteristics, including neural fate commitment, apical-basal polarity establishment, and interkinetic nuclear migration (IKNM) (Shi et al., 2012a; Shi et al., 2012b; Zhu et al., 2013). To observe the effect of high glucose in our *in vitro* models, we examined all three characteristics in both models under different glucose conditions.

**FIGURE 1:**
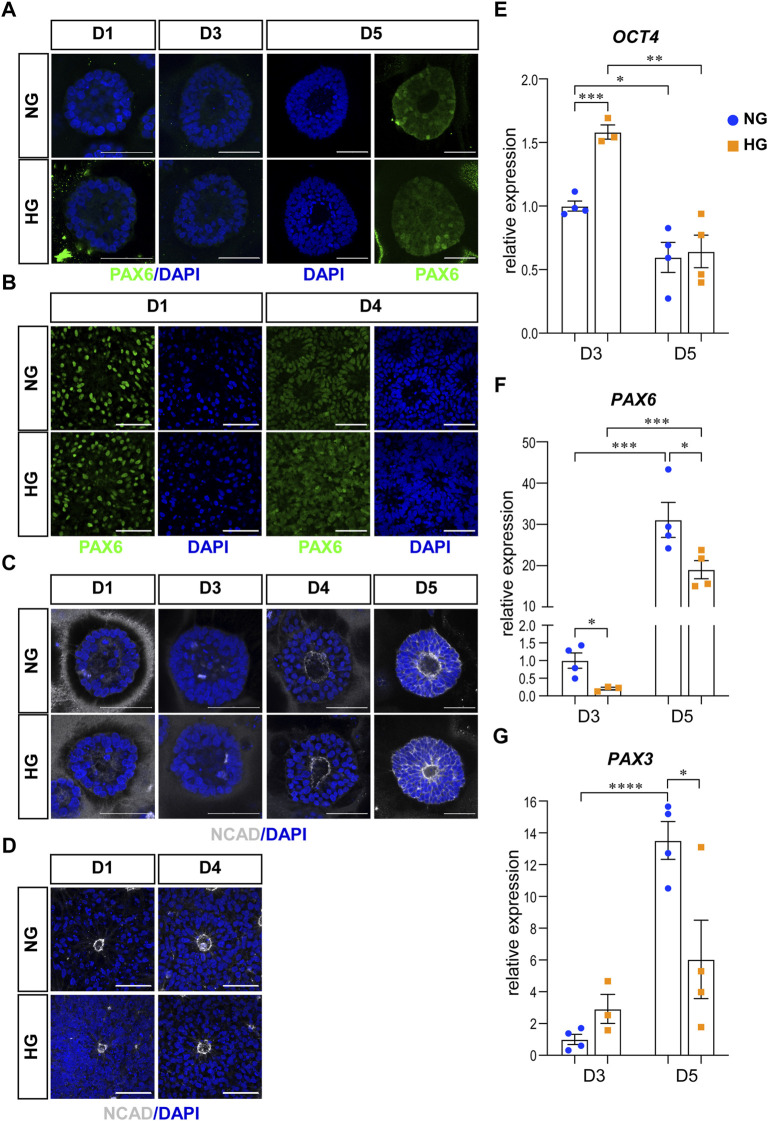
High glucose did not affect differentiation or polarity in either models. **(A)** Immunofluorescence images of PAX6 (green) in the normal (NG) and 2x high glucose (HG) groups at D1, 4, and 5 of the 3D neuroepithelial cysts. **(B)** PAX6 (green) staining in the normal (NG) and 2x high glucose (HG) groups at D1 and D4 after passage in rosette model. **(C)** Immunofluorescence images of NCAD (grey) at D1, 3, 4, and 5 of the 3D neuroepithelial cysts. **(D)** NCAD (grey) staining at D1 and D4 after passage in rosette model. All scale bar = 50 μm. 3D neuroepithelial cysts were collected at either D3 or D5. Total RNA was extracted and subjected for qRT-PCR. N ≥ 3 wells/condition. The relative expression level of each gene was calculated to compare to the expression level in NG group at D3. The results for OCT4 **(E)**, PAX6 **(F)**, PAX3 **(G)** were presented in **(E–G)** respectively.

The first characteristic is the neural fate commitment, which indicates a change in cell potential from totipotency to neural stemness. Therefore, we examined the change of OCT4, PAX6, and NCAD in our models. OCT4 is a totipotency marker and the expression of OCT4 can be observed during the stem cell stage in our models. As neural induction went on, the expression of OCT4 decreased and eventually disappeared at the end of the culture in both models. PAX6 and NCAD are two neural progenitor markers. In the 3D neuroepithelial cyst model, the expression of PAX6 can first be observed at D5 (Figure 1A), while the expression of NCAD can be observed at D4 and was elevated at D5 (Figure 1C). qRT-PCR experiments confirmed that high glucose did not affect the decrease of OCT4 (Figure 1E) and the increase of PAX6 expression at D5 (Figure 1F). However, at D3 the high glucose treatment slowed the differentiation, as the expression of OCT4 was higher at D3 (Figure 1E) and PAX6 was lower at D3&D5 in high glucose group than in the normal group (Figure 1F). It has been reported that PAX3 is down-regulated by high glucose in differentiated mouse neural stem cells in either neurospheres culture (Yang et al., 2016) or neuroepithelial cysts culture (Sanders et al., 2014). Therefore, the expression of PAX3 was also quantified in our 3D models by qRT-PCR experiments. The expression of PAX3 was significantly increased at D5 compared to D3 and it was lower in high glucose group than that in the normal glucose group at D5 (Figure 1G). In the rosette model, both PAX6 and NCAD were induced during the neural induction stage and thus can be observed in cells at D1 after passage (Figures 1B, D).

The second characteristic is the establishment of apical-basal polarity. After polarity establishment, apical maker proteins including those associated with adherens junctions are concentrated at the apical plasma membrane (Escuin et al., 2015). One of the adherens junction proteins, NCAD is also an apical marker for apical-basal polarity. In our models, the expression of NCAD concentrated at the center of the 3D neuroepithelial cyst model and the rosette model. This distribution marked an inner apical polarity, which is similar to that in the *in vivo* neural tube and the established *in vitro* models (Wu et al., 2021). The distributions of NCAD were not affected by high glucose in either model system (Figures 1C, D).

**FIGURE 2:**
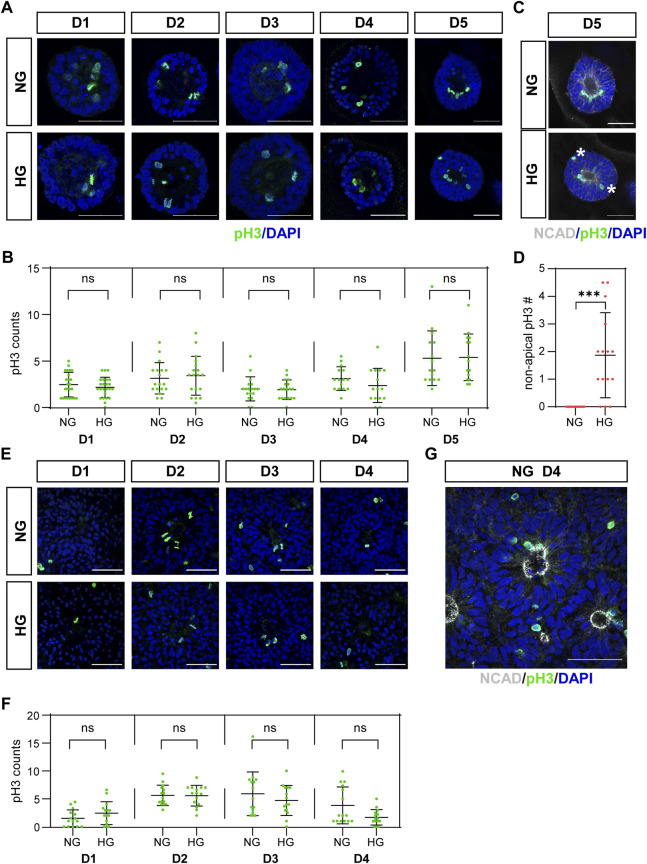
**(C)** High glucose disrupted apical location of pH3 positive cells in the 3D model but not in the rosette model.pH3 (green) staining was performed in both models. The representative images were shown in **(A)** for the 3D cyst model and **(E)** for the rosette model. The number of pH3-positive dividing cells was presented in **(B,F)**, respectively. N ≥ 15/condition. **(C)** Double immunofluorescence images of pH3 (green) and NCAD (grey) at D5 of the cysts. Asterisks mark dividing nuclei located at non-apical positions. The statistical analysis of apical and non-apical located pH3 positive cells at D5 of neuroepithelial cysts were presented in **(D)** N = 15/condition**. (G)** Double immunofluorescence images of pH3 (green) and NCAD (grey) at D4 after passage in the rosette model. NG, normal glucose. HG, 2X high glucose. DAPI (blue) shows the nucleus. All scale bar = 50 μm.

The third characteristic is the IKNM in which the nuclei of dividing cells only appear at the apical side. The nuclei of dividing cells in our models were labeled with anti-pH3 antibody and observed by immunofluorescence. As the results showed, almost all pH3 positive cells were near the center of the 3D cysts or 2D rosettes, which are on the apical side (Figures 2A, C, E, G). We noticed that pH3 positive nuclei were observed at non-apical positions in the high glucose group at D5 in the 3D neuroepithelial cyst model (Figure 2C, labeled by stars), which was not observed in the control group (Figures 2C, D). We did not observe there are some non-apical pH3 positive nuclei even in the normal glucose condition in the rosette model (Figures 2E, G) and there is no obvious differences on the apical-basal position of dividing nuclei in the rosette model between two glucose conditions (Figure 2E).

We also counted the number of mitotic cells labeled with pH3. The results showed that high glucose did not increase the number of mitotic cells at all examined time points in both models (Figures 2B, F).

### High glucose increased cell apoptosis in both models

Previous studies in animal models have shown that apoptosis is involved in high glucose-induced NTDs at the cellular level (Loeken, 2020). We also checked apoptosis in our models by immunofluorescence using the apoptosis marker CASP3. The density of CASP3 was used to evaluate the level of apoptosis in our models and compared between two glucose conditions.

**FIGURE 3:**
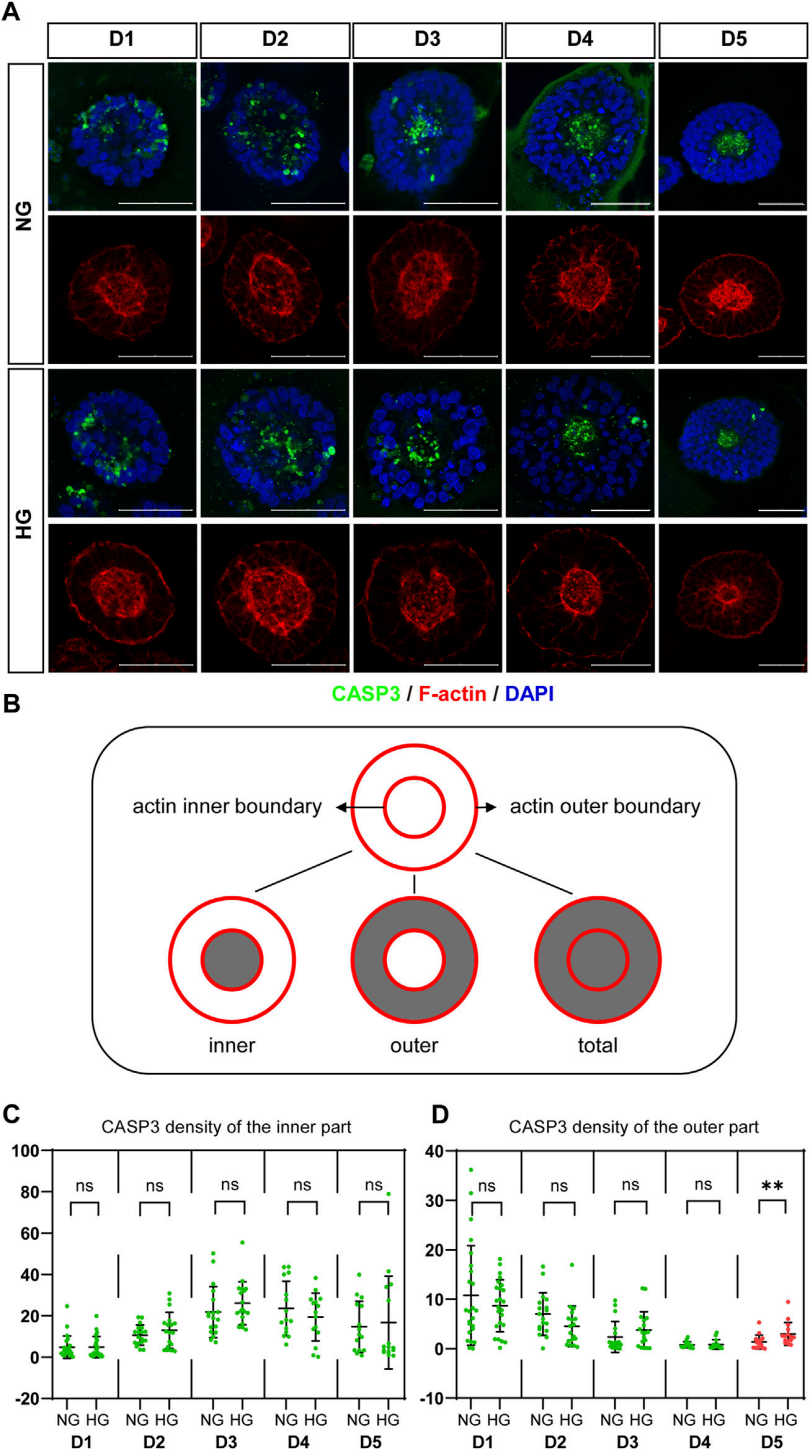
High glucose increases the apoptosis at the end of 3D model culture. **(A)** Double immunofluorescence images from CASP3 (green) and F-actin (red) in the normal (NG) and 2x high glucose (HG) groups in D1-5. DAPI (blue) shows the nucleus. Scale bar is 50 μm. **(B)** Illustration of F-actin stained images. The red lines represent the actin inner and outer boundaries. The area enclosed by actin inner boundary is named “inner” area. The area enclosed by actin outer boundary is named “total” area. The “outer’ area is the total area subtracted the inner area, which is occupied by cells. CASP3 density in the inner **(C)** or the outer **(D)** areas of the 3D models at D1-5 were calculated. N ≥ 15/condition.

**FIGURE 4:**
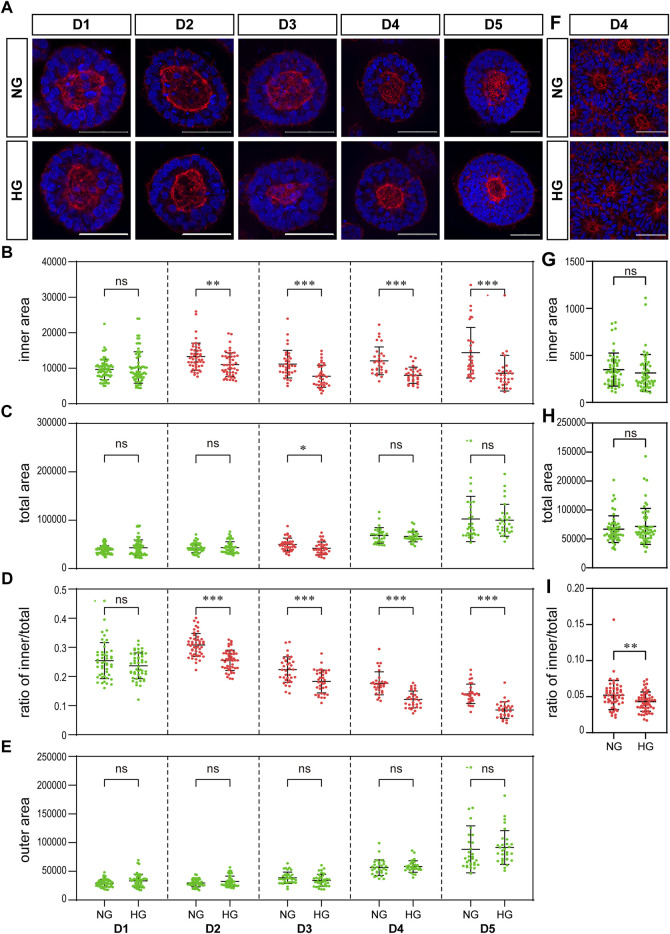
High glucose results in a significant reduction in the actin inner boundary enclosed area in the 3D model and the ratio of inner to total area in the rosette model. **(A)** Representing F-actin (red) immunofluorescence images in the normal (NG) and 2x high glucose (HG) groups at D1-5 of neuroepithelial cysts. DAPI (blue) shows the nucleus. Scale bar is 50 μm (**B–E)**. The inner area enclosed by the actin inner boundary **(B)** and the total area enclosed by actin outer boundary **(C)** were measured by ImageJ software. The ratio of the inner to total area **(D)** and the outer area occupied by cells **(E)** were calculated. N ≥ 30/condition. **(F)**. Representing F-actin (red) staining images in the normal (NG) and 2x high glucose (HG) groups at D4 after passage of the rosette model. DAPI (blue) shows the nucleus. All scale bar = 50 μm. The inner area enclosed by the actin inner boundary **(G)** and the total area enclosed by actin outer boundary **(H)** were measured by ImageJ software, and the ratio of the inner to total area **(I)** were calculated. N ≥ 30/condition.

CASP3 was co-stained with F-actin. We observed that actin is highly concentrated at both the apical and basal edges of the 3D cysts, which forms an inner and an outer boundary respectively. Cells inside the cyst are radially arranged between the two boundaries (Figure 3A; Figure 4A). Therefore, we defined different areas of each cyst based on actin (Figure 3B) and measured the density of CASP3 in each cyst. The results showed that, the density of CASP3 in the cysts, which are between the actin inner and outer boundary, was significantly higher under high glucose condition in the 3D model at D5 (Figure 3D). There was some high CASP3 signal inside the cysts (Figure 3A), but the density was not changed by high glucose condition (Figure 3C). In the rosette model, the density of CASP3 was also significantly higher under high glucose condition at post-passage D4 (Supplementary Figure S1). Therefore, 2x high glucose increased apoptosis in both models at the end stage of the culture. This is consistent with the previous studies in animal models.

### High glucose results in a significant reduction in the ratio of actin inner boundary enclosed area to outer boundary enclosed area

We examined the effect of high glucose on actin in our two models. The distribution of actin showed similar characteristics in both models. That is, actin is highly concentrated at both the apical and basal edges of the 3D cysts and the rosettes, which forms an inner and an outer boundary respectively (Figures 4A, F).

We noticed that the area enclosed by the actin inner boundary was significantly smaller in the high glucose group than that in the normal glucose group in the 3D model from D2 onwards (Figure 4B). At the same time, there was no significant difference in the total area enclosed by actin outer boundary between two glucose conditions on most time points (Figure 4C). We calculated the area occupied by cells (“outer area”) and the ratio of actin inner to outer boundaries enclosed areas (“ratio of inner/total”). This ratio of inner/total areas was also significantly smaller in the high glucose group than that in the normal group from D2 onwards (Figure 4D). At the same time, the area occupied by cells (outer area) were not changed (Figure 4E), In the rosette model, the actin outer boundary of the rosette structure appears at D4 at latest. Therefore, we only measured the areas enclosed by actin boundaries at D4. There is no significant difference in the areas enclosed by either inner or outer boundaries between the two glucose conditions (Figures 4G, H). However, when we calculated the ratio of inner to total areas, it was also significantly smaller in the high glucose group than in the control group (Figure 4I), which is similar to the 3D model.

**FIGURE 5:**
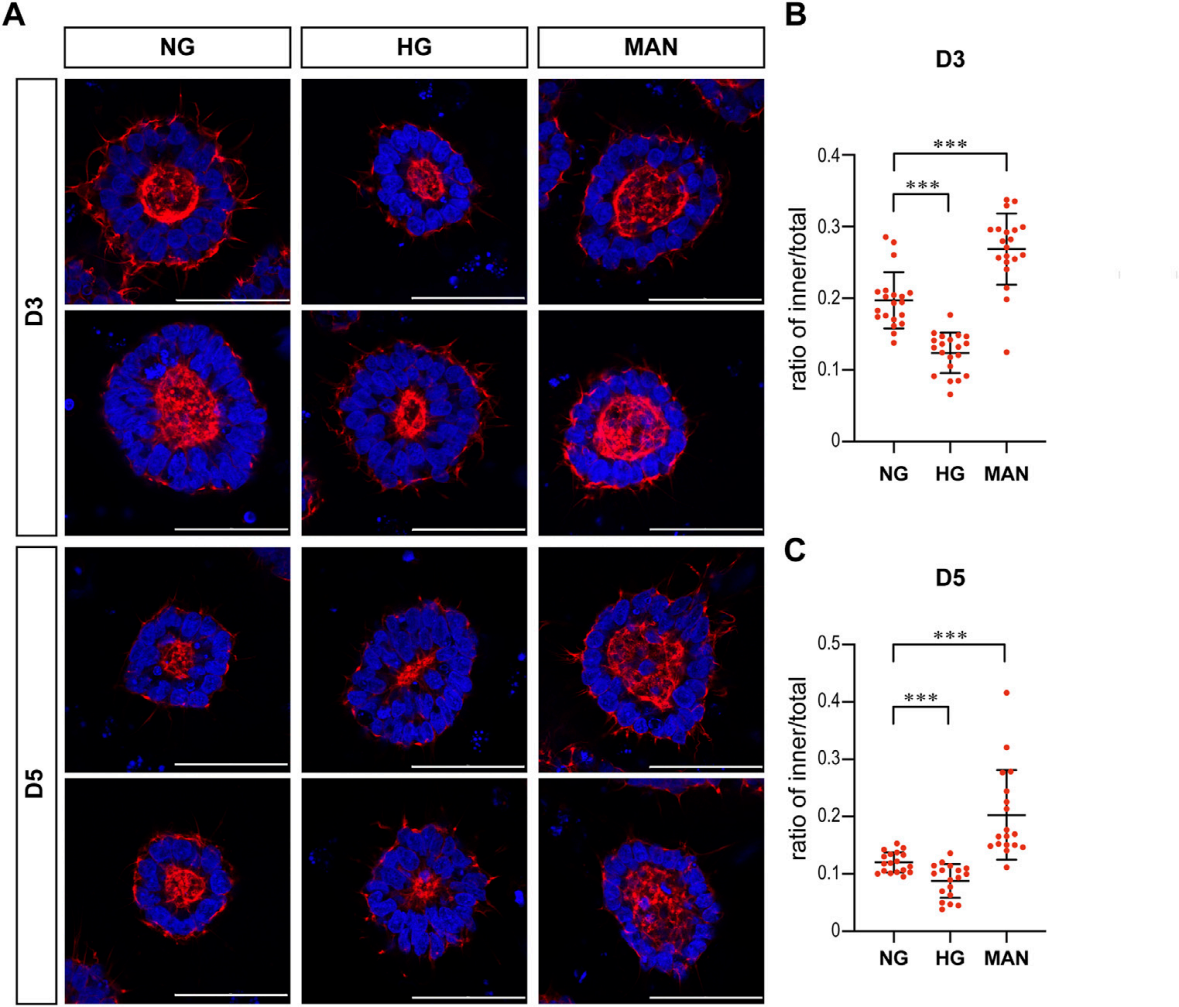
Equimolar mannitol did not reduce the actin inner boundary in the 3D models. **(A)** Representing F-actin (red) immunofluorescence images in the normal (NG), 2x high glucose (HG), and equimolar mannitol groups at D3 & D5. DAPI (blue) shows the nucleus. Scale bar is 50 μm (**B,C)**. The ratio of the inner to total area were calculated for each group and the statistical data for D3 or D5 were shown in **(B)** and **(C)** respectively. N ≥ 18/condition.

To eliminate the effect of osmolarity changes caused by the 2X glucose, we also tested whether equimolar mannitol would have the same effect on actin. As shown in Figure 5, equimolar mannitol did not reduce the ratio of inner/total areas, but rather increased this ratio.

### High glucose causes a change in HK1 distribution

As osmolarity is unlikely to be the cause of the reduced inner actin boundary, we next examined several actin binding proteins and catalytic enzymes which may affect actin dynamic in our models. Firstly, we performed immunofluorescence to observe pMLC and pCFN in our models as actin can be regulated by pMLC and pCFN during neural tube development (Nikolopoulou et al., 2017). There was no obvious difference in either the expression or distribution of pMLC and pCFN between the two glucose groups (Supplementary Figure S2). Next, we focused on carbohydrate metabolic enzymes in our models since it has been known that carbohydrate metabolic enzymes can affect actin as well (DeWane et al., 2021). There are five rate-limiting enzymes of central carbohydrate metabolism that are located in the cytoplasm, which are hexokinase (HK), phosphofructokinase (PFK), pyruvate kinase (PK), lactate dehydrogenase (LDH), and glucose 6-phosphate dehydrogenase (G6PD). Based on the Bgee database (Bastian et al., 2021), the isoenzymes of these metabolic enzymes that are more likely to be expressed during neural tube development were chosen and examined in our models, which are HK1, PFKM, PKM2, G6PD, and LDHB. LDHA and LDHB are two subunits of LDH. LDHA may preferentially catalyze pyruvate to lactate, while LDHB prefers the reverse reaction (Doherty and Cleveland, 2013). Therefore, both subunits were included in the following experiments.

**FIGURE 6:**
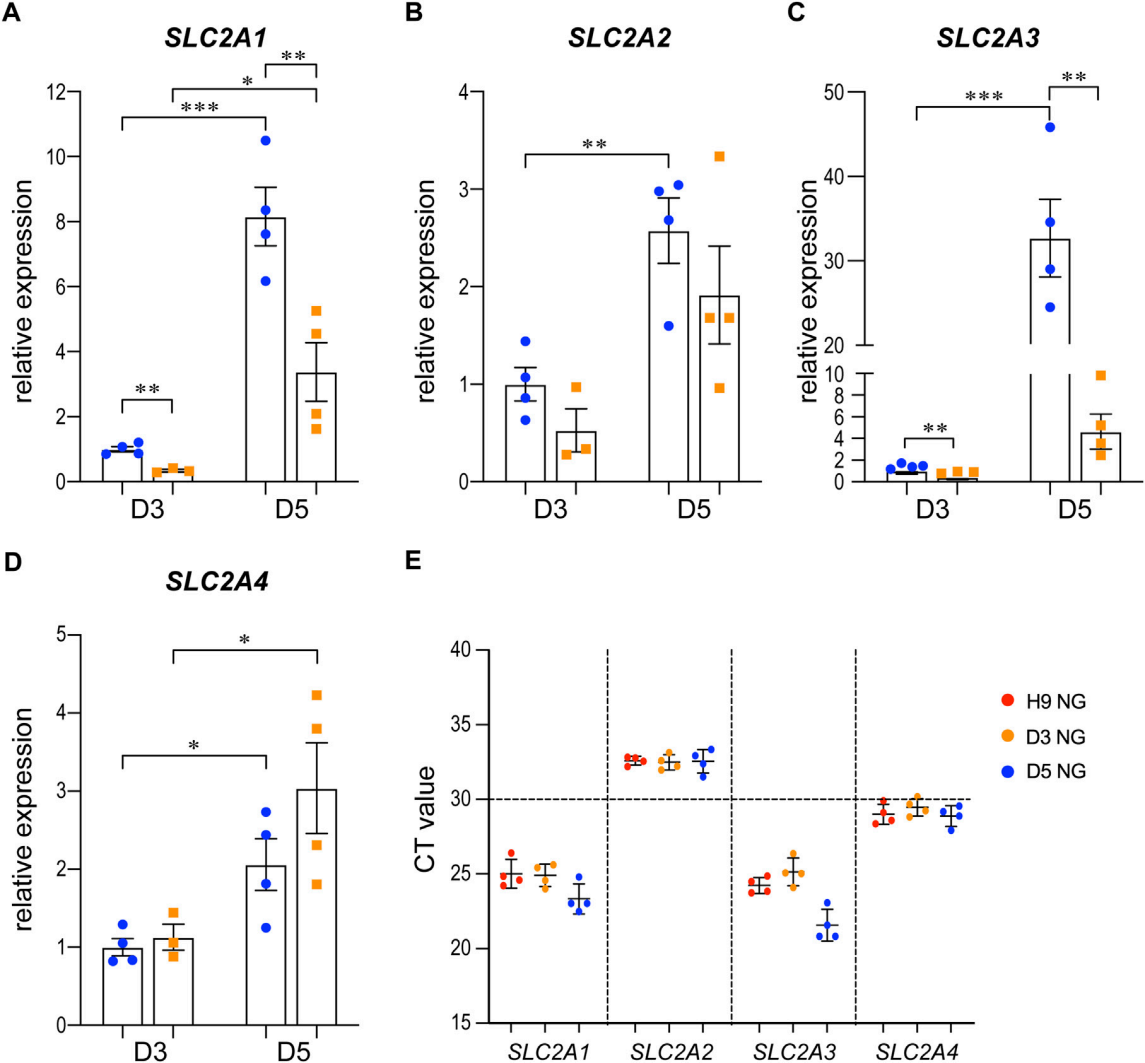
The expression changes of SLC2A1-4 in control and high glucose groups of the 3D models at D3 and D5.3D neuroepithelial cysts were grown in either normal glucose (NG) or 2x high glucose (HG) conditions. Cysts were collected at either D3 or D5. Total RNA was extracted and subjected for qRT-PCR. N ≥ 3 wells/condition. The relative expression level of each gene was calculated to compare to the expression level in NG group at D3. The results for SLC2A1-4 were presented in **(A–D)** respectively. The CT values of qRT-PCR for these four genes in the H9 cells and cysts at either D3 or D5 from the NG groups were shown in **(E)**.

**FIGURE 7:**
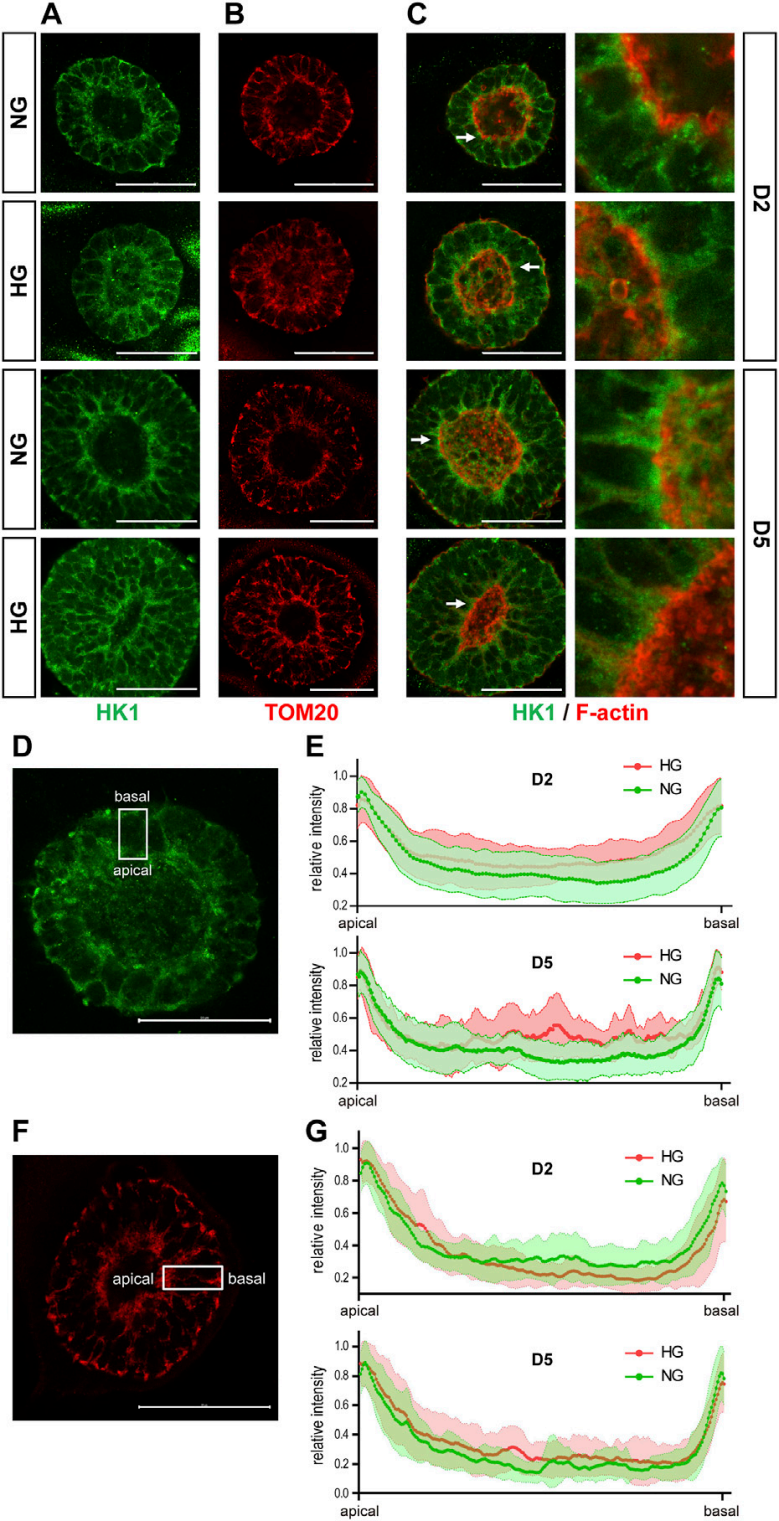
High glucose causes HK1 distribution change in the 3D model. **(A)** Representing images of HK1 (green) in normal (NG) and 2x high glucose (HG) groups at D2 and D5. **(B)** Representing TOM20 (red) staining at D2 and 5. **(C)** Double immunofluorescence images of HK1 (green) and F-actin (red) at D2 and D5. All scale bar is 50 μm. **(D)** Illustration of HK1 distribution. **(E)** The line profile plots for HK1 distribution relative to the peak intensity at D2 and D5. (N ≥ 9/condition). **(F)** Illustration of TOM20 distribution. **(G)** The line profile plots for TOM20 distribution relative to the peak intensity at D2 and D5. N ≥ 6/condition. All scale bar = 50 μm.

We examined the expression of *SLC2A1*, 2, *3*, and *4*, which are the genes that encode the glucose transporters GLUT1-4, by qRT-PCR in the 3D cysts. The results showed that high glucose significantly inhibited the upregulation of *SLC2A1* and *SLC2A3* at D5 (Figure 6A, C) but not had no obvious effect on *SLC2A2* or *SLC2A4* (Figures 6B, D). To be noticed, both GLUT2 and GLUT4 have very low expression levels in H9 cells and neuroepithelial cysts derived from H9 cells as indicated by the CT value (Figure 6E). As previous studies suggest that GLUT2 is the essential glucose transporter for diabetes associated NTDs in mouse models (Li et al., 2007), we further tested the expression of GLUT2 in the following immunofluorescence experiment. We examined the expression of seven proteins by immunofluorescence in both models. Particular attention was paid to the 3D model at two time points, D2 and D5. We did not observe any differences in either the expression or distribution of six out of seven proteins between the two glucose groups (Supplementary Figure S3), with an exception of HK1. In the 3D model, the distribution of HK1 was more condensed and highly distributed on the apical and basal surfaces in the control glucose group at both D2 and D5 (Figure 7A; Figures 7D, E). Under high glucose conditions, HK1 was more diffused and had less obvious apical-basal distribution tendency at D5 (Figures 7A, E). Higher magnification images showed that HK1 was next to the actin boundary but not co-localized (Figure 7C). In the 2D model, HK1 was also mainly distributed on the apical and basal surfaces of the rosette structure (Supplementary Figure S4A). High glucose did not cause a change in this apical-basal distribution in the rosette model, but the density of HK1 was lower in the high glucose group than in the control group (Supplementary Figure S4A). We also examined the distribution of HK1 and actin together in the rosettes. The results showed that HK1 was also located close to actin at the inner boundary but they were not co-localized (Supplementary Figure S4C).

HK1 can be located at either the cytoplasm or outer mitochondrial membrane (Pastorino and Hoek, 2008). Previous studies have found that the product of HK, glucose-6-phosphate, inhibits the mitochondrial localization of HK (Wilson, 1995). Therefore, it is possible that the altered HK1 distribution in the 3D model and the reduced HK1 density in the rosette model may result from the reduced mitochondrial localization of HK1 induced by high glucose levels. To test this hypothesis, we examined the distribution of mitochondria in our models with TOM20 (translocase of outer membrane 20 kDa subunit), a mitochondrial outer membrane protein. In the 3D model, the distribution of TOM20 is also condensed on the apical and basal edges (Figures 7B, F, G). Such distribution is not affected by high glucose (Figures 7B, G). The distribution of HK1 was similar to that of TOM20 in the normal glucose group; while the distribution of HK1 was less similar to that of TOM20 in the high glucose group at D5 (Figures 7A, B; Figures 7E, G). In the rosette model at post-passage D4, both HK1 and TOM20 were mainly located on the apical and basal side of the rosette and their distributions were not affected by high glucose in the rosette model (Supplementary Figures S4A, B).

## Discussion

By using neuroepithelial cysts and rosettes models, we investigated the effect of high glucose on these models. Beside increased apoptosis, we observed that the apical location of pH3 labeled dividing nuclei was disrupted by high glucose. High glucose also caused a relatively smaller actin inner boundary enclosed area, which is unlikely due to the osmolarity effect of high glucose. The actin and HK1 were next to each other but not co-localized and high glucose caused a change in the HK1 distribution in the 3D model. These results suggested that the effect high glucose is much broader than just inducing cell apoptosis during neural tube development.

Our first observation is the non-apical location of pH3 labeled dividing nuclei in the 3D model under high glucose conditions. Such abnormal location of pH3 labeled dividing nuclei suggested an abnormal IKNM happened under the high glucose condition. However, further experiments with live-imaging method will be needed to confirm whether there are abnormal movements of dividing nuclei in these models. Further, whether abnormal IKNM happens or not *in vivo* under high glucose conditions will also need further investigation.

Another interesting observation is that high glucose caused smaller actin inner boundary enclosed area in our 3D models. Equal molarity application of mannitol did not have the same effect on our 3D models, suggesting the effect of high glucose on actin is probably not due to osmolarity changes. As actin is essential for cell shape changes and neural tube development, our observation suggested that high glucose might be able to cause abnormal neural tube development through actin. Similar to what we observed in our models, actin is accumulated apically in the developing neural tube (Suzuki et al., 2012). Therefore, if apical actin is also affected by high glucose *in vivo*, it might lead to a change in neural tube morphology. The change on actin happens earlier than in the non-apical location change of pH3 labeled dividing nuclei and increased apoptosis, suggesting the effects of high glucose on actin are earlier events. Whether there is any link between actin dynamic changes and abnormal nuclei migration or apoptosis will need further study.

The next question is why high glucose can cause such changes in actin. Although previous studies showed that GLUT2 has high kinetic to glucose and is the essential glucose transporter for diabetes associated NTDs in mouse models (Li et al., 2007), we observed very low expression of GLUT2 in our models and its expression was not changed by high glucose condition. Whether this is due to the difference between human and mouse; and furthermore whether GLUT2 is also the major glucose transporter for diabetes associated NTDs in human will need further investigation. Based on previous reports, actin is regulated by actin binding proteins MLC and CFN in the developing neural tube (Suzuki et al., 2012; Nikolopoulou et al., 2017). Both proteins are regulated by their phosphorylation state, so we checked pMLC and pCFN in our models, but no significant difference was observed between high and normal glucose groups. It is possible that we did not observe the phosphorylation state changes of MLC and CFN in our models as we only examined our model at D5. More detailed observation with live image method may be needed in the future. On the other hand, metabolic changes support the energy requirements of actin dynamics in the context such as mechanotransduction, cell migration, or epithelial to mesenchymal transitions (DeWane et al., 2021). Here, we observed that HK1 located next to inner boundary actin and high glucose changed the HK1 distribution. Whether HK1 distribution changes is related to actin changes under high glucose condition will needs further investigation in the future.

Our models are human stem cells based and easy-to-operate. They can mimic some aspects of the neural tube development such as cellular differentiation and apical basal polarity. However, our organoid-like methods still have several limitations. First of all, our 3D model starts from a cluster of neural progenitor cells to form a cyst structure with a lumen inside. Such a process resembles more closely the secondary neurulation process instead of primary neurulation in which the neural tube closure actually happens. To our knowledge, there is no current method that can mimic the actual neural tube closure process *in vitro*. Therefore, live-imaging on whole embryos from the mouse or rat is still needed in the future to observe the effects of high glucose on actin dynamic during primary neurulation. In addition, our model do not have dorsal-ventral patterning. More matured models will be needed in the future. Secondly, although we observed many similar features in both the 3D cysts and the rosette models, they still have some differences. For example, we did not observe the pH3-labeld nuclei were abnormally located in the rosette model under high glucose condition. This is probably due to the fact that each rosette structure is connected in the rosette model, while each cyst is isolated in the 3D model. Therefore, the 3D organoid-like model is probably more sensitive than the rosette model as we only observed the HK1 distribution change by high glucose in 3D model but not in the rosette model. Last but not least, the glucose concentration in our control group is already higher than the physiological blood glucose concentration. However, the glucose concentration used in all current methods for human stem cells based neural epithelial cysts and brain organoids is much higher than the physiological condition (Zhu et al., 2013; Lancaster and Knoblich, 2014; Ogura et al., 2018; Duval et al., 2019; Zheng et al., 2019; Tang et al., 2022). It is possible such high glucose is required for the organoid’s survival *in vitro*. However, methods for human stem cells based neural epithelial cysts or organoids grow in physiological glucose concentration will be needed in the future, just like what have been done for mouse stem cells based models (Sanders et al., 2014).

Nonetheless, by using a sensitive 3D neuroepithelial cysts model and a rosette model, we observed that high glucose can cause actin dynamic changes in very early time point and abnormally non-apical located dividing cells at a later time point. Such observation broadened our view for the cellular effects of high glucose on neural tube development, especially on secondary neurulation process.

## Data Availability

The original contributions presented in the study are included in the article/Supplementary Material, further inquiries can be directed to the corresponding author.

## Supplementary material

SUPPLEMENTARY FIGURE S1 High glucose increased the apoptosis at the end of rosettes culture. **(A)** Immunofluorescence images of CASP3 (green) in the normal (NG) and 2x high glucose (HG) groups at D1-4 after passage. DAPI (blue) shows the nucleus. All scale bar is 50 μm. **(B)** The density of CASP3 staining were measured by Image J software. *N*=15/condition.

SUPPLEMENTARY FIGURE S2 High glucose had no obvious effect on the distribution of pMLC and pCFN in both models. Double immunofluorescence images of F-actin (red) and pMLC (green) (**A,B)** or pCFN (green) **(C,D)** were performed in the normal (NG) and 2x high glucose (HG) groups. (**A,C)** are from 3D cyst models at D1, 2, 5. **(B,D)** are from 2D rosette models at D3,4 after passage. N≥6/condition. DAPI (blue) shows the nucleus. All scale bar = 50 μm.

SUPPLEMENTARY FIGURE S3 High glucose had no obvious effect on the distribution of GLUT2, PFKM, PKM2, LDHA, LDHB, and G6PD in the 3D model. Double immunofluorescence images of F-actin (red) and GLUT2 (green) , **(A)** PFKM (green) **(B)**, PKM2 (green) **(C)**, LDHA (green) **(D)**, LDHB (green) **(E)**, or G6PD (green) **(F)** were performed on the 3D models at D2 and 5 from both the normal (NG) and 2x high glucose (HG) groups. N ≥ 6/condition. DAPI (blue) shows the nucleus. Scale bar is 50 μm.

SUPPLEMENTARY FIGURE S4 HK1 distribution in the rosette model. Representing images of **(A)** HK1 (green), **(B)** TOM20 (red), and **(C)** double staining of HK1 (green) and F-actin (red) in normal (NG) and 2x high glucose (HG) groups at D4 after passage. N ≥ 6/condition. All scale bar = 50 μm.
