# Supplementary material for: High glucose causes developmental abnormalities in neuroepithelial cysts with actin and HK1 distribution changes
Source: Front Cell Dev Biol. 2023 Jan 6;10:1021284. doi: 10.3389/fcell.2022.1021284 (PMC9852901; doi:10.3389/fcell.2022.1021284)
Supplement: Supplementary file 1 [file Table1.DOCX]

**Table S1 Antibodies used in our experiments**

| **Category** | **Antigen** | **Dilution ratio** | **Company & Catalog#** |
| --- | --- | --- | --- |
| **Primary antibody** | NCAD | 1:200 | abcam, ab19348 |
|  | pH3 | 1:500 | abcam, ab32388 |
|  | cleaved caspase3 | 1:400 | Cell Signaling Technology,9661 |
|  | PAX6 | 1:100 | proteintech, 12323-1-AP |
|  | pMLC | 1:100 | Cell Signaling Technology,3671 |
|  | pCFN | 1:50 | Cell Signaling Technology,3313 |
|  | GLUT2 | 1:100 | proteintech, 20436-1-AP |
|  | HK1 | 1:400 | proteintech, 19662-1-AP |
|  | PFKM | 1:100 | proteintech, 55028-1-AP |
|  | PKM2 | 1:100 | proteintech, 15822-1-AP |
|  | LDHA | 1:50 | proteintech, 19987-1-AP |
|  | LDHB | 1:400 | proteintech, 14824-1-AP |
|  | G6PD | 1:400 | proteintech, 25413-1-AP |
|  | TOM20 | 1:500 | proteintech, 66777-1-Ig |
| **Secondary antibody** | Goat anti-Rabbit IgG (H+L) Cross-Adsorbed Secondary Antibody, Cyanine5 | 1:500 | Invitrogen, A10523 |
|  | Goat Anti-Rabbit IgG H&L (Alexa Fluor® 488) preadsorbed | 1:500 | abcam, ab150081 |
|  | Goat Anti-Mouse IgG H&L (Alexa Fluor® 647) | 1:500 | abcam, ab150115 |
|  | Goat Anti-Mouse IgG H&L (Alexa Fluor® 488) preadsorbed | 1:500 | abcam, ab150117 |
|  | Rhodamine Phalloidin | 1:500 | Invitrogen, R415 |
|  | DAPI | 1:1000 | Beyotime, C1002 |
