# Supplementary figures and images for: High glucose causes developmental abnormalities in neuroepithelial cysts with actin and HK1 distribution changes
Source: Front Cell Dev Biol. 2023 Jan 6;10:1021284. doi: 10.3389/fcell.2022.1021284 (PMC9852901; doi:10.3389/fcell.2022.1021284)

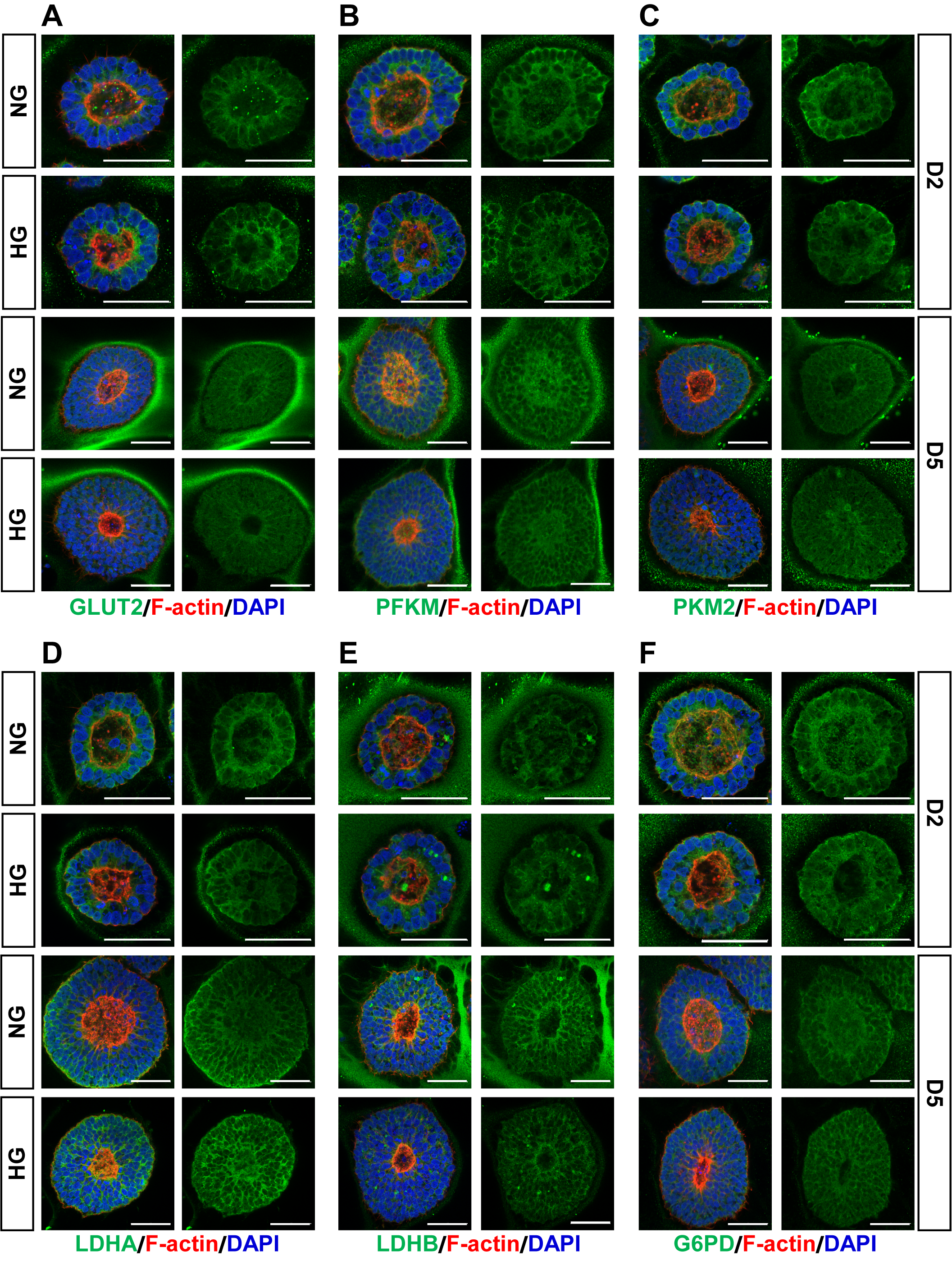

Supplement: Supplementary file 2 [file Image3.TIF]

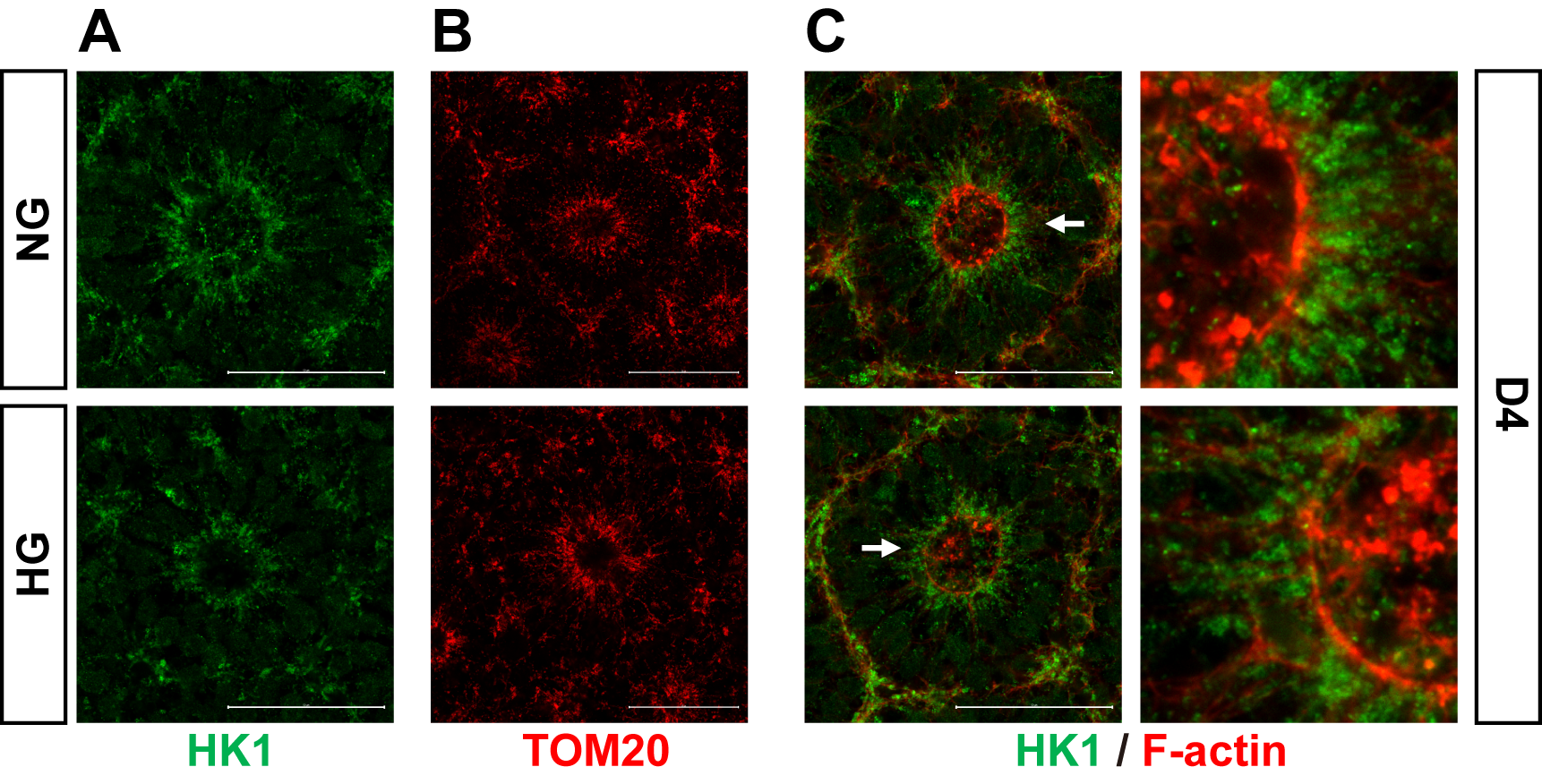

Supplement: Supplementary file 3 [file Image4.TIF]

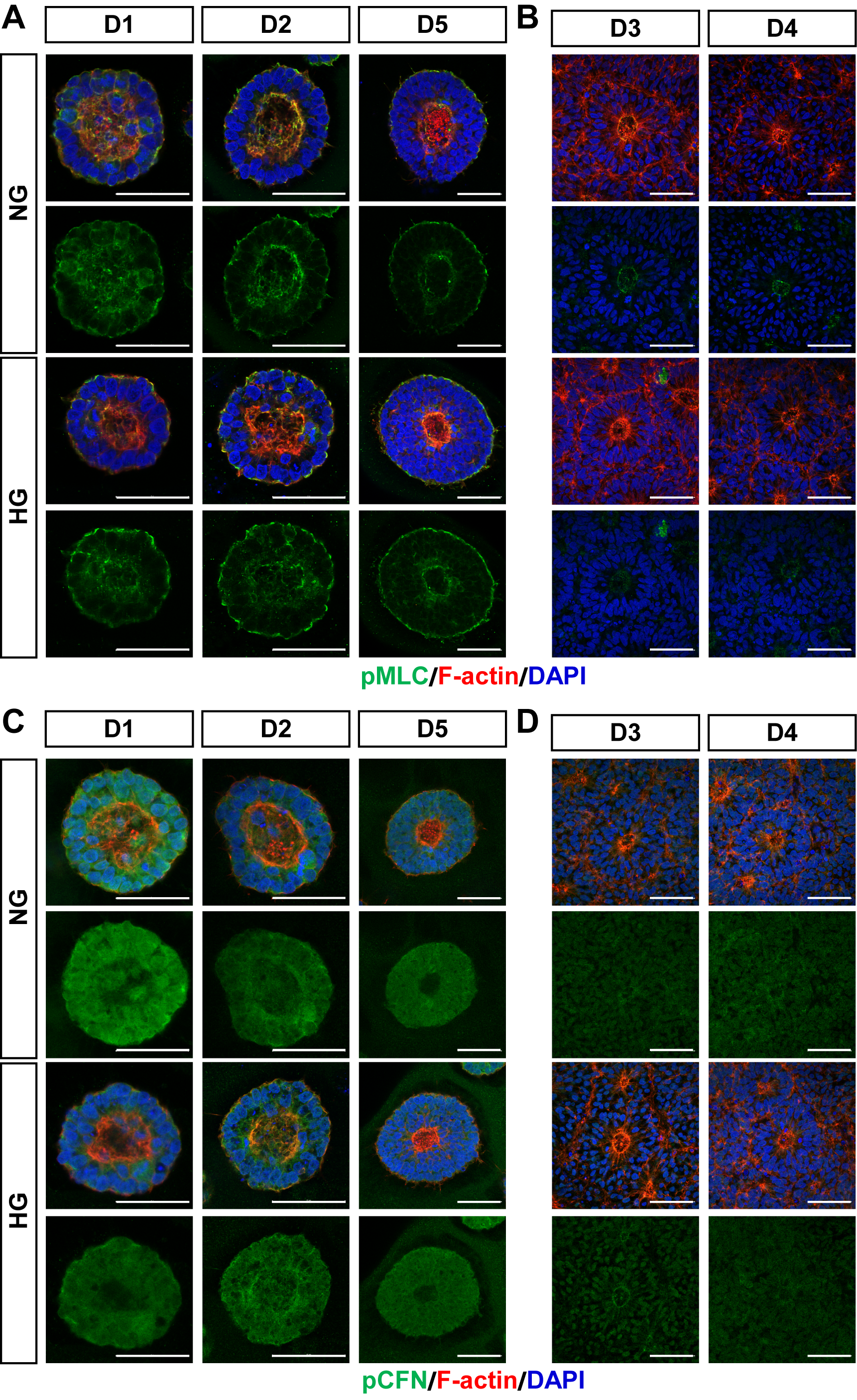

Supplement: Supplementary file 4 [file Image2.TIF]

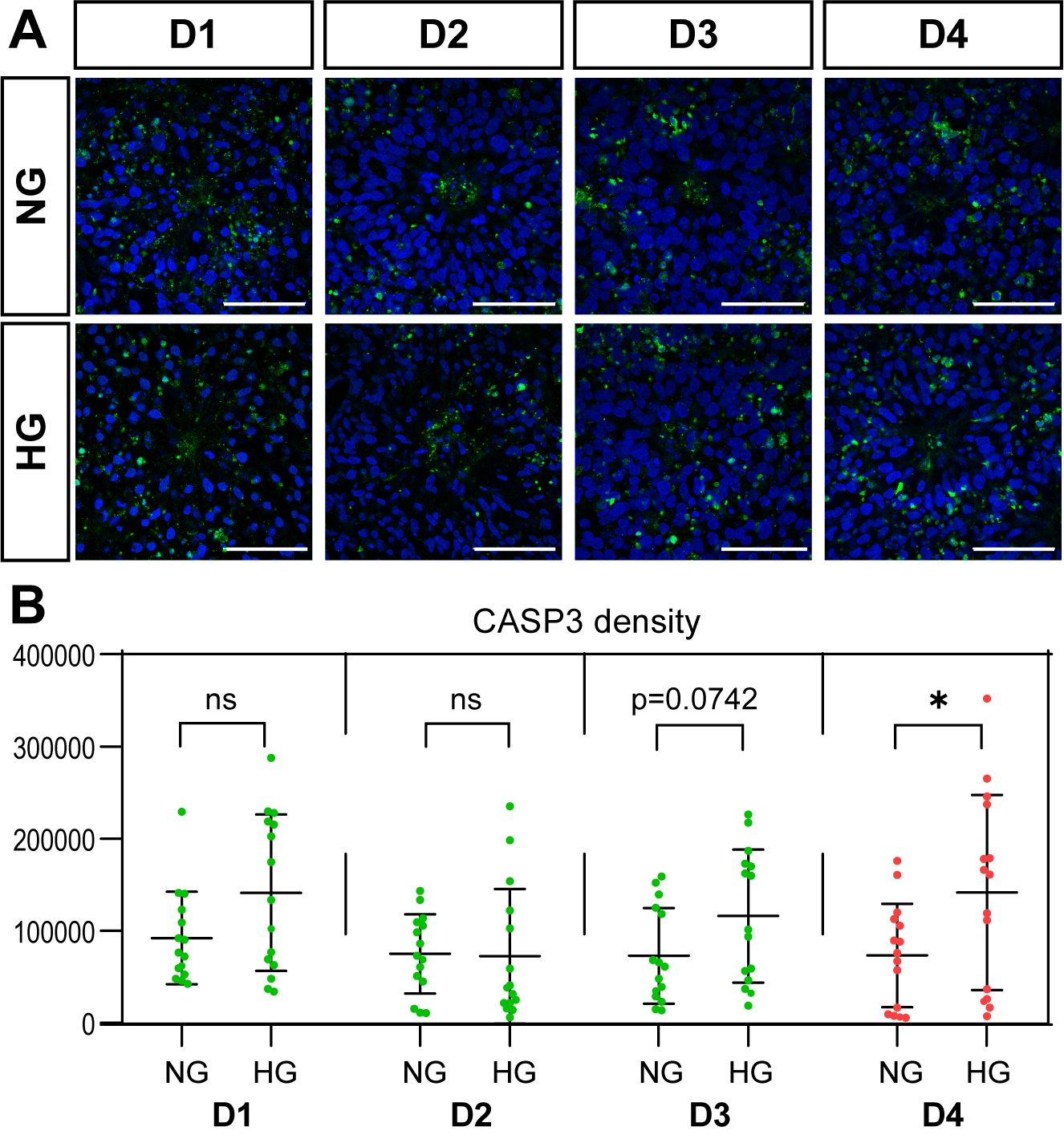

Supplement: Supplementary file 5 [file Image1.TIF]
