# Supplementary material for: High glucose causes developmental abnormalities in neuroepithelial cysts with actin and HK1 distribution changes
Source: Front Cell Dev Biol. 2023 Jan 6;10:1021284. doi: 10.3389/fcell.2022.1021284 (PMC9852901; doi:10.3389/fcell.2022.1021284)
Supplement: Supplementary file 6 [file Table2.DOCX]

**Table S2. Primers for qRT-PCR analyses**

| **Gene Name** | **Forward Primer 5’-3’** | **Reverse Primer 5’-3’** |
| --- | --- | --- |
| *SLC2A1* | ATTGGCTCCGGTATCGTCAAC | GCTCAGATAGGACATCCAGGGTA |
| *SLC2A2* | AACCCAAAACCAACCCCTTG | TTCCACCAACTGCAAAGCTG |
| *SLC2A3* | GCTGGGCATCGTTGTTGGA | GCACTTTGTAGGATAGCAGGAAG |
| *SLC2A4* | GCTGGGCTTCTTCATCTTCAC | AGTTCTGTGCTGGGTTTCAC |
| *PAX3* | AGCTCGGCGGTGTTTTTATCA | CTGCACAGGATCTTGGAGACG |
| *OCT4* | CAAAGCAGAAACCCTCGTGC | TCTCACTCGGTTCTCGATACTG |
| *PAX6* | TGGGCAGGTATTACGAGACTG | ACTCCCGCTTATACTGGGCTA |
| *ACTB* | CATGTACGTTGCTATCCAGGC | CTCCTTAATGTCACGCACGAT |
